# Meta-analysis of the relation between European and American smokeless tobacco and oral cancer

**DOI:** 10.1186/1471-2458-7-334

**Published:** 2007-11-15

**Authors:** Rolf Weitkunat, Edward Sanders, Peter N Lee

**Affiliations:** 1 PMI Research & Development, Philip Morris Products S.A., Neuchâtel, Switzerland; 2 P.N. Lee Statistics and Computing Ltd, Surrey, UK

## Abstract

**Background:**

Smokeless tobacco is often referred to as a major contributor to oral cancer. In some regions, especially Southeast Asia, the risk is difficult to quantify due to the variety of products, compositions (including non-tobacco ingredients) and usage practices involved. In Western populations, the evidence of an increased risk in smokeless tobacco users seems unclear, previous reviews having reached somewhat differing conclusions. We report a detailed quantitative review of the evidence in American and European smokeless tobacco users, and compare our findings with previous reviews and meta-analyses.

**Methods:**

Following literature review a meta-analysis was conducted of 32 epidemiological studies published between 1920 and 2005 including tests for homogeneity and publication bias.

**Results:**

Based on 38 heterogeneous study-specific estimates of the odds ratio or relative risk for smokeless tobacco use, the random-effects estimate was 1.87 (95% confidence interval 1.40–2.48). The increase was mainly evident in studies conducted before 1980. No increase was seen in studies in Scandinavia. Restricting attention to the seven estimates adjusted for smoking and alcohol eliminated both heterogeneity and excess risk (1.02; 0.82–1.28). Estimates also varied by sex (higher in females) and by study design (higher in case-control studies with hospital controls) but more clearly in studies where estimates were unadjusted, even for age. The pattern of estimates suggests some publication bias. Based on limited data specific to never smokers, the random-effects estimate was 1.94 (0.88–4.28), the eight individual estimates being heterogeneous and based on few exposed cases.

**Conclusion:**

Smokeless tobacco, as used in America or Europe, carries at most a minor increased risk of oral cancer. However, elevated risks in specific populations or from specific products cannot definitely be excluded.

## Background

Oral cancer, histologically most frequently squamous-cell carcinoma, includes malignant neoplasms of the lip, tongue, palate, gum, piriform sinus, floor of the mouth, pharynx, tonsils, salivary glands and unspecified parts of the mouth [1]. It is the eighth and 14^th ^most incident cancer worldwide in men and women, respectively [2]. Age-standardised mortality rates (per 100,000 per year) differ regionally, ranging (in 2001) from 2.4 in Sweden to 21.2 in Hungary. Trends differ also, with, in the past two decades, decreases in the US, Finland and Sweden, and increases in Hungary, the Czech Republic, Germany and Norway [3,4]. US mortality rates are higher in blacks than whites, and prevalence is greater in areas with a high proportion of Asians [5].

Among specific risk factors commonly discussed are alcohol, solar radiation, genetic predisposition, and tobacco (smoking and smokeless). Smoking has been estimated to pose twice as high a risk of oral cancer as does smokeless tobacco use [6]. Except for the exact magnitude of the risk difference, this is rarely questioned, and usually attributed to the lack of combustion products from smokeless tobacco [7]. Smokeless tobacco products are traditionally classified as snuff or chewing tobacco [8]. In Europe and North America usage is mostly oral, while nasal use of finely ground "dry snuff" has become rare [9]. In the US, finely cut "moist snuff" or chewing tobacco is held (or in the case of the latter chewed) in the gingival buccal area. In Scandinavia snuff (or snus in Sweden) is generally placed under the upper or lower lip.

Oral tobacco has long been referred to as a major contributor to oral cancer incidence. In line with International Agency for Research on Cancer [IARC] monograph 37 [10], the US Surgeon General [11] concluded in 1986 that "the association between smokeless tobacco use and cancer is strongest for cancers of the oral cavity" and that "evidence for an association between smokeless tobacco use and cancers outside the oral cavity in humans is sparse." The conclusion for oral cancer was based on only a few, mainly quite small studies that provided quantitative data.

Later Gross et al. [12] meta-analysed 12 US studies conducted between 1952 and 1993. They computed a random-effects relative risk of 1.74 (95% confidence interval [CI]: 1.32 to 2.31) and concluded that "a relative risk < 2.0 may be considered to represent a weak association because of the biases and confounders that tend to affect observational studies." While the individual relative risk [RR] estimates ranged from 0.99 to 4.44, those in 12 studies in Southeast Asia ranged from 2.2 to 39.19, corresponding to an overall estimate of 8.94 (95% CI: 5.26–15.18). Gross et al. concluded that "the studies in Southeast Asia suggest a strong relationship between the risk of oral cancer and the use of chewing tobacco. The tobacco chewed in these countries was often mixed with some other substances, such as betel quid and areca nut. It is still unclear whether it is the tobacco or the substance added [that] plays the major role." The authors also reported estimates from four studies in other regions, two in Europe and two in Latin America. These ranged from 0.67 to 1.40, more resembling the US than the Southeast Asian findings.

In 1996 Pershagen [9] published a wide-ranging review on exposure and health aspects of smokeless tobacco. With respect to oral cancer he stated that "methodological limitations in the studies make it difficult to interpret the findings." Nevertheless, based on six case-control studies from Sweden and the US, and with particular emphasis on the study of Winn et al. [13], which he considered to be the most conclusive, Pershagen inferred that "habitual use of oral tobacco can increase the risk of oral cancer." He cited an odds ratio [OR] of 4.2 for the Winn et al. study. However, this estimate refers to only a subgroup (white nonsmoking women). In contrast, the estimate based on all the available data was lower at 2.7 (1.8–3.9).

In a toxicological and epidemiological assessment of the health risks of snuff dipping, Nilsson [14] concluded that "although a small risk cannot be excluded, the use of smokeless tobacco products low in TSNA which now are available on the market entails a risk that at any rate is more than 10 times lower than that associated with active smoking." He pinpointed "a wide discrepancy" between the estimated cancer risk found in studies conducted in Sweden and the US. The US data were drawn mostly from the Winn et al. study [13]. For Sweden Nilsson concluded that although about "20% of all grown-up Swedish males use moist snuff, it has not been possible to detect any significant increase in the incidence of cancer of the oral cavity or pharynx – the prevalence of which by international standards remains low in this country." At the ecological level the situation is similar in the US. West Virginia has the highest consumption of smokeless tobacco of all US states, but below average oral cancer incidence rates [15].

Rodu and Cole [16] conducted a meta-analysis on smokeless tobacco use and upper respiratory tract cancer based on 21 publications. Although the available data about tobacco type are sparse and ambiguous in some reports, the authors attempted separate analyses. For oral and pharyngeal cancer they estimated a relative risk of 1.1 (0.8–1.6) for chewing tobacco, 0.7 (0.4–1.2) for moist snuff, 4.0 (2.7–5.9) for dry snuff and 1.5 (1.1–2.0) for unspecified smokeless tobacco use. They concluded that "the use of moist snuff and chewing tobacco imposes minimal risks for cancers of the oral cavity and other upper respiratory sites..."

Critchley and Unal [17] conducted a narrative literature review of studies with a total sample size of at least 500 participants, including studies in both Western and Southeast Asian countries. They concluded that the study of Winn et al. [13] "remains the strongest evidence for an association of ST use with oral cancers in the US, although it is limited to women and was carried out many years ago." They based this conclusion on the same OR estimate of 4.2 that Pershagen [9] cited. For Scandinavian studies the reviewers concluded that "although these findings are consistent with no effect, the studies do not have sufficient power to detect a moderately raised OR." Overall, according to Critchley and Unal, there exists "...a substantial risk of oral cancers in India" while for the US and Scandinavia "most recent studies...are not statistically significant, but moderate positive associations cannot be ruled out due to lack of power."

The information we examined suggests at most a minor increased risk of oral cancer associated with smokeless tobacco use in Western countries. Elsewhere, products used are likely to differ in toxicity, and their associated risk, especially in Southeast Asia, is difficult to quantify due to the variety of products, compositions (including non-tobacco ingredients) and usage practices involved. Our objective was to conduct a detailed analysis of published evidence on the association of American and European smokeless tobacco and oral cancer, and to calculate a pooled effect estimate. Compared with the reviews of Rodu and Cole [16] and Critchley and Unal [17] we aimed to include more studies, and carry out a more detailed quantitative analysis, including random-effects and fixed-effect meta-analyses and an investigation of heterogeneity.

## Methods

### Study identification and selection

RW identified reports from a systematic search, not limited by period or language, of MEDLINE, EMBASE, CANCERLIT and TOXLINE through March 2005. The main searches used combinations of the terms "smokeless tobacco", "chewing tobacco", "snuff", and "snus" for exposure, and "oral cancer" and "mouth neoplasms" for outcome. Specific oral cancer sites corresponding to ICD-10 codes C00 to C14 [1] were also searched for. Further articles were identified from reference lists in individual papers and reviews.

All reports had to satisfy the following *Inclusion criteria*: published in peer reviewed journal or publicly available, based on research on humans, of cohort or case-control design, study location specified, any form of oral cancer as the outcome, and chewing tobacco, orally used moist snuff or unspecified smokeless tobacco as the exposure. They also had to satisfy the *Exclusion criteria*: sample included in a more complete or recent study, conducted in an Asian population, no control group, inappropriate design (case report, qualitative study or review/meta-analysis), insufficient power (less than five expected exposed cases), appropriate risk estimates and CIs not reported and cannot be computed from the available data.

Having completed this process, RW then compared the selected studies with those of a previous unpublished review conducted in 2002 by PNL based on MEDLINE, reference lists and reviews.

### Data extraction

From each report details were abstracted relating to the study (design, period, region, population, sample selection, size, and matching criteria), the exposure (method of assessment, type of smokeless tobacco investigated, exposure doses and durations considered), the outcome (type and location of oral cancer and method of diagnosis), and issues relating to analysis (type of effect measure, analysis methods, stratification variables, and adjustment factors).

To avoid bias, it would be advantageous to restrict attention to estimates of effect size (OR or RR) and of precision (95% CI) based on subjects who have never smoked. However, such results are rarely available, and are often based on very limited numbers of cases. Our main analyses are therefore based on estimates derived from the whole population, smokers and nonsmokers combined, except for studies where the only available results are specifically for smokers or for nonsmokers. However, estimates of effect size and precision specifically for never smokers were also extracted for an additional analysis (see the end of the results section).

Where possible, separate estimates were obtained for male and female subjects and for different types of smokeless tobacco; chewing, snuff or smokeless (unspecified or chewing/snuff). Estimates for ever exposure were preferred to estimates for current exposure. Adjusted estimates were preferred to crude estimates and where multiple adjusted estimates were available, that adjusted for the most potential confounding variables was used.

In many studies, the required estimates of effect size and precision were not given by the authors and had to be estimated. Crude estimates were derived from the relevant 2 × 2 table using standard methods, with, where necessary, numbers estimated from proportions given numerically or graphically and/or combined over types of oral cancer or levels of exposure. Independent crude estimates were combined over strata by fixed-effect meta-analysis [18] to obtain estimates adjusted for the stratifying variables. Non-independent adjusted estimates were combined over exposure levels or types of oral cancer using a method that accounts for the correlation of estimates with a common baseline group [19-21]. In a few studies where information was available on chewing and on snuff but not on joint use, approximate estimates for combined smokeless use were obtained assuming that no-one used both products.

### Meta-analysis

For effect size estimate, the standard error of its logarithm was calculated from its reported or estimated CI, assuming that the effect size was log-normally distributed. The logarithms of the effect sizes and their corresponding standard errors formed the data points for fixed-effect and random-effects meta-analyses [18]. Separate sets of meta-analyses were carried out for chewing tobacco, for snuff and for overall smokeless tobacco use (both from studies where only estimates for unspecified use were available and from studies where the estimate was derived from the separate data for chewing tobacco and snuff). A fourth set of meta-analyses, referred to as "all" types in the tables, used the smokeless tobacco estimates where available and otherwise the estimates specifically for chewing tobacco or for snuff. Within each set, meta-analyses were carried out based on all the estimates, regardless of the extent of confounding adjustment, and on estimates that were unadjusted or adjusted for various combinations of smoking, alcohol, age and social status. For each analysis, within-group heterogeneity was assessed by the chi-squared test of homogeneity [22] and by the I^2 ^statistic [23].

For selected meta-analyses, a forest plot is shown. Each estimate is shown as a rectangle, with its area proportional to its weight. The CI is indicated by a horizontal line. The data are plotted on a logarithmic scale so that the estimate is centred in the CI. Also shown in the plot are the actual values of the estimate and its CI and weight. Results from a random effects meta-analysis are also shown. The combined estimate is presented as a diamond with the width corresponding to the CI, and the estimate as the centre of the diamond.

For the "all" types set of estimates, sensitivity analyses were carried out by eliminating those estimates that were only for smokers or only for nonsmokers, and also by replacing those estimates that were calculated approximately assuming that there were no joint users of chewing tobacco and snuff by estimates for chewing tobacco in US studies and for snuff in Swedish studies. Heterogeneity was also investigated further, separately for estimates unadjusted for any factor and estimates adjusted for smoking. Subgroups were defined *a priori *by type of smokeless tobacco, sex, study design, study location, study period, and by whether or not the estimate was reported by the author or was derived by us. Between-subgroup heterogeneity was assessed by a chi-squared test [24]. To describe the contribution of the investigated subgroups further, the between-subgroup chi-squared was expressed as a percentage of the total chi-squared.

Additional meta-analyses were carried out for the set of estimates for smokeless tobacco use among never smokers.

Publication bias was assessed by a funnel plot, in which the logarithm of the effect size was plotted against its precision [25,26].

## Results

1313 potentially relevant studies were identified by the literature search, but only 63 appeared possibly of value from initial assessment. These underwent more detailed examination and 32 met the inclusion and exclusion criteria. Of the 31 studies that did not, ten were excluded due to insufficient quantitative information [14,27-35], five to lack of a control group [36-40], two to limited power[41,42], three to the outcome not being oral cancer [43-45], and three to non-Western smokeless tobacco [46-48]. Two reports [49,50] were letters commenting on studies already rejected for other reasons, and two were reviews [51,52]. Four publications [53-56] referred to studies which were more recently or more completely or adequately covered in other publications [13,57-59].

### Extracted data

Table 1, sorted on publication year, gives details of the 29 case-control and three cohort studies considered in this analysis. The reports were published between 1920 and 2005. 25% of the studies were published up to 1970 and 25% after 1994. Most studies (no = 24) were conducted in the USA, with three in Sweden (studies 19, 27, 29), and one each in Puerto Rico (study 7), the UK (study 9), Brazil (study 18), Denmark (study 26), and Norway (study 31).

**Table 1:** Study overview (included studies).

**Study**	**First author, year of publication**	**Region**	**Study period**^a^	**Design**^b^	**Sex**	**Case definition (or at risk population**^c^**)**^d^	**Controls (or years of follow-up**^c^**)**	**Matching factors**^e^
**1**	Broders 1920 [74]	USA	up to 1920	CCH	M+F	NS	No lip cancer	NS
**2**	Moore 1953 [57]	USA	1951–1952	CCH	M	50+ years	No cancer	NS
**3**	Wynder 1957 [58]	USA	up to 1957	CCH	M^f^	NS	Benign diseases, lymphoma, skin and gastrointestinal cancer	Age, rel, ins, ses
**4**	Peacock 1960 [93]	USA	1952–1958	CCH	M+F	With data on tobacco habits	No oral cancer	Sex, race, ins
**5**	Vogler 1962 [62]	USA	1956–1957	CCH	M+F	Incident and prevalent	No oral cancer	None
**6**	Vincent 1963 [94]	USA	up to 1963	CCH	M^g^	Incident	Successive gastrointestinal patients	Age
**7**	Martinez 1969 [95]	Puerto Rico	1966	CCH/P	M+F	Incident in 1966	Patients without cancer and community controls	Age, sex
**8**	Keller 1970 [75]	USA	1958–1962	CCH	M	Discharged with complete records	No oral cancer	Age, hosp, time
**9**	Browne 1977 [96]	UK	1957–1971	CCP	M+F	NS	Community controls	Age, sex, occ, area
**10**	Williams 1977 [97]	USA	1969–1971	CCH	M+F	Incident, Third National Cancer Survey	No tobacco- or alcohol-related cancers	None
**11**	Wynder 1977 [98]	USA	1969–1975	CCH	M	Histologically confirmed	No tobacco-related diseases	Age, race, area
**12**	Westbrook 1980 [63]	USA	1955–1975	CCH	F	Incident	Unstated hospital controls	Age, time
**13**	Winn 1981 [13]	USA	1975–1978	CCH	F	Incident and death certificate	No oral cancer or mental disease	Age, race, type, area
**14**	Wynder 1983 [99]	USA	1977–1980	CCH	M^f^	Histologically confirmed	No tobacco-related diseases	Age, race, hosp, ins
**15**	Stockwell 1986 [65]	USA	1982	CCP	M+F	Cancer registry 1982	Melanoma or endocrine cancer	None
**16**	Blot 1988 [100]	USA	1984–1985	CCP	M+F	Incident	Population controls	Age, sex, area, race
**17**	Spitz 1988 [101]	USA	1985–1987	CCH	M+F	Registered 1985–1986, completed questionnaire, histologically confirmed with squamous cell carcinoma of defined sites	No squamous cell carcinoma	Age, sex
**18**	Franco 1989 [102]	Brazil	1986–1988	CCH	M+F	Incident, not lip or salivary glands	No cancer or mental disorder	Age, sex, area, time
**19**	Blomqvist 1991 [103]	Sweden	up to 1991	CCH	M+F	NS	No cancer	Age, sex
**20**	Maden 1992 [104]	USA	1985–1989	CCP	M	Squamous cell, 18–65 years, with residential telephone	Population controls	Age, time
**21**	Sterling 1992 [67]	USA	1986	CCP	M+F	Registered in National Mortality Followback Study 1986, age 25+ years	Population controls	None
**22**	Zahm 1992 [72]	USA	1954–1980	Cohort	M	US veterans who returned questionnaire on tobacco use in 1954 or 1957	Mortality 1954–1980	NA
**23**	Mashberg 1993 [68]	USA	1972–1983	CCH	M	Incident in 1972–1983, US veterans	No cancer or dysplasia of pharynx, larynx, lung or oesophagus	None
**24**	Perry 1993^h^	USA	about 1992	CCH	M+F	NS	Cardiovascular diseases	NS
**25**	Kabat 1994 [59]	USA	1977–1990	CCH	M+F	Incident	No tobacco-related diseases	Age, sex, race, hosp, time
**26**	Bundgaard 1995 [105]	Denmark	1986–1990	CCP	M+F	Histologically confirmed	Population controls	Age, sex, area
**27**	Lewin 1998 [64]	Sweden	1988–1990	CCP	M	Incident and registered, not identified at autopsy	Population controls	Age, area
**28**	Muscat 1998 [106]	USA	1977–1990	CCH	M+F	Incident, not mentally impaired	No tobacco-related diseases	Age, sex, race, time
**29**	Schildt 1998 [73]	Sweden	1980–1989	CCP	M+F	Histologically confirmed, not deceased without a relative	Population controls	Age, sex, area, vit
**30**	Schwartz 1998 [69]	USA	1990–1995	CCP	M+F	Histologically confirmed squamous cell carcinoma, 18–65 years	Population controls	Age, sex
**31**	Boffetta 2005 [82]	Norway	1966–2001	Cohort	M	Population sample and relatives of migrants to USA	Incidence 1966–2001	NA
**32**	Henley 2005 [66]	USA	1959–2000	Cohort	M	Never smokers in Cancer Prevention Study I (CPS I) and II (CPS II)	Mortality CPS I : 1959 to 1972 CPS II : 1982 to 2000	NA

^a ^In studies 3 and 6 the starting year of collection of cases was not given

^b ^CCH = case control with hospital controls, CCP = case control with population controls

^c ^For cohort studies

^d ^NS = not stated

^e ^NS = not stated, NA = not applicable, rel. = religion, ins. = insurance status, hosp. = hospital, time = period of admission, occ. = occupation, type = incident or decedent, vit. = vital status

^f ^Exposure data only available for males

^g ^Insufficient information for females

^h ^Attributable oral cancer risk due to smokeless tobacco use based on a case-control study at Sinai Hospital in Detroit; unpublished

Not all studies were primarily concerned with the relationship between smokeless tobacco and oral cancer. Consequently, study-specific inclusion and exclusion criteria varied, as did criteria for matching of controls. Of the 29 case-control studies, seven (studies 3, 10, 11, 14, 15, 25, 28) selected controls without smoking-related diseases, five (2, 7, 18, 19, 23) selected controls without any cancer or without oral and other specific cancers, nine (1, 4, 5, 6, 8, 12, 13, 17, 24) selected patient controls without oral cancer, and eight (9, 16, 20, 21, 26, 27, 29, 30) selected population controls essentially without restriction.

Table 2 shows the cancer sites considered in the 32 studies. The definitions of sites were often imprecisely stated, so the table must be regarded as an approximation.

**Table 2:** Cancer sites considered in the 32 included studies

**Cancer site**	**Study numbers**																															
	1	2	3	4	5	6	7	8	9	10	11	12	13	14	15	16	17	18	19	20	21	22	23	24	25	26	27	28	29	30	31	32

Buccal mucosa		X	X	X	X				X			X													X	X						
Floor of mouth		X	X	X	X				X						X			X		X					X	X				X		
Gingiva					X																				X							
Gum/palate		^a^	X		X				X	X		X			X			X		X					X	X			X	X		
Lip	X	^a^	X		X			X		X					X				X	X									X			
Oral cavity/mouth			X			X	X			X	X		X	X		X	X	X			X	X	X	X	X	X	X		X	X	X	X
Pharynx/alveolus		X	X	X	^b^	X	X		X	X			X	X	X	X	X			X	X	X	X		X	X	X			X	X	X
Tongue		^a^	X		X					X					X		X	X		X					X	X			X	X		
Tonsils		^a^	X																						X					X		
Salivary glands					X					X					X													X				
Unspecified					^b^				X						X		X^c^			X				X						X		

^a ^Specifically excluded by author

^b ^Due to the joint reporting of pharynx and larynx cases, both sites were excluded from analyses

^c ^Larynx cases could not be excluded due to incomplete reporting

Table 3 contains the main results (i.e. those for smokers and nonsmokers combined where possible) for the 32 studies. Further estimates relating to the site where the smokeless tobacco was held are given elsewhere[60]. Most studies contrasted never and ever use of smokeless tobacco and did not specify any minimal duration of use, but three studies (2, 4, 7) considered only exposures of 20 years or more. Separate estimates by gender were only available in five studies (4, 7, 10, 16, 25). Estimates were only available for chewing tobacco in four studies (3, 7, 9, 26), for snuff in six studies (6, 12, 13, 19, 27, 31) and for unspecified smokeless tobacco use in 12 studies (2, 4, 8, 10, 15, 16, 18, 20, 22, 24, 30, 32). Estimates were available for more than one type of product in the remaining 10 studies (1, 5, 11, 14, 17, 21, 23, 25, 28, 29), all but two (5, 25) including an estimate for overall smokeless tobacco use. Most of the estimates were for smokers and nonsmokers combined. Exceptionally, that for chewing tobacco, in study 3, was for smokers, those for chewing tobacco in study 7 were for nonsmokers, those for snuff in studies 19 and 25 were for nonsmokers, and those for smokeless tobacco in study 32 were for nonsmokers. Two effect estimates based on different data sets (the cancer prevention studies CPS-I and CPS-II) were provided by study 32.

**Table 3:** Study details and effect estimates.

**Study**	**First author, year of publication**	**Method of exposure assessment**	**Cancer site**	**Adjustment factors**^a^	**Exposure (period)**	**Sex**	**Cases/Controls**^b^	**OR/RR**^c^**(95% CI)**
**1**	Broders 1920 [74]	NS	Lip	Smoking	Chewing (NS)	M+F	537/500	2.05 (1.48–2.83)^de^
				None	Snuff (NS)	M+F	537/500	1.76 (0.12–26.2)^ef^
				None	Smokeless (NS)	M+F	537/500	2.05 (1.48–2.83)^efg^
**2**	Moore 1953 [57]	Interview	Buccal mucosa, gum, floor of mouth	None	Smokeless (20+ years)	M	112/38	3.00 (1.37–6.54)^f^
**3**	Wynder 1957 [58]	Interview	Lip, floor of mouth, gum, buccal mucosa, tongue, palate, tonsil, pharynx	Smoking^h^	Chewing (ever)	M	525/186	2.00 (1.16–3.47)^ef^
**4**	Peacock 1960 [93]	Interview	Buccal mucosa, alveolar ridge, floor of mouth	Age, ins.	Smokeless (20+ years)	M	25/191	3.06 (1.08–8.63)^di^
						F	20/172	2.00 (0.66–6.01)^di^
**5**	Vogler 1962 [62]	Interview or questionnaire	Lip, buccal mucosa, tongue, palate, gingiva, floor of mouth, minor salivary glands^k^	None	Chewing (ever)	M	14/402	7.38 (4.31–12.62)^dij^
					Snuff (ever)	F	75/826	38.28 (21.49–68.15)^dei^
**6**	Vincent 1963 [94]	Interview	Oral cavity, pharynx	None	Snuff (NS)	M	66/100	4.22 (1.41–12.63)^fi^
**7**	Martinez 1969 [95]	Interview	Mouth and pharynx	Smoking^l^	Chewing (last 20 years)	M	18/81	2.29 (0.62–8.48)^fim^
						F	16/79	0.34 (0.04–2.79)^fim^
**8**	Keller 1970 [75]	Hospital records	Lip	Smoking	Smokeless (NS)	M	258/207	3.63 (1.02–12.95)^d^
**9**	Browne 1977 [96]	Interview, proxy interview	Buccal mucosa, upper alveolus and hard palate, lower alveolus, floor of mouth, pillar of fauces, soft palate, unspecified or multiple	None	Chewing (NS)	M+F	75/150	0.67 (0.27–1.66)^f^
**10**	Williams 1977 [97]	Interview	Lip, tongue, salivary glands, gum, mouth, pharynx	None	Smokeless (ever)	M	190/1788	0.91 (0.53–1.56)^fgi^
						F	79/3188	1.54 (0.37–6.42)^fgi^
**11**	Wynder 1977 [98]	Interview	Oral cavity	None	Chewing (ever)	M	591/2560	1.15 (0.85–1.55)^f^
					Snuff (ever)	M	591/2560	0.62 (0.32–1.22)^f^
					Smokeless (ever)	M	591/2560	1.02 (0.78–1.34)^fgn^
**12**	Westbrook 1980 [63]	Hospital records	Buccal mucosa, gum	None	Snuff (ever)	F	55/55	540.0 (60.97–4782.82)^f^
**13**	Winn 1981 [13]	Interview, proxy interview	Mouth and pharynx	Smoking, race	Snuff (ever)	F	232/410	2.67 (1.83–3.90)^d^
**14**	Wynder 1983 [99]	Interview	Oral and pharyngeal	None	Chewing (ever)	M	414/414	1.00 (0.62–1.61)^ef^
					Snuff (ever)	M	414/414	0.42 (0.11–1.65)^f^
					Smokeless (ever)	M	414/414	0.90 (0.57–1.41)^efgn^
**15**	Stockwell 1986 [65]	Medical records	Lip, tongue, salivary glands, gum, floor of mouth, other parts of mouth, pharynx	None	Smokeless (ever)	M+F	1462/8285	2.02 (1.01–4.02)^fi^
**16**	Blot 1988 [100]	Interview, proxy interview	Oral and pharyngeal	None	Smokeless (ever)	M	762/837	0.85 (0.57–1.26)^ef^
						F	352/431	3.44 (1.09–10.91)^ef^
**17**	Spitz 1988 [101]	Questionnaire	Larynx, tongue, orohypopharynx, oral cavity^o^	None	Chewing (ever)	M+F	185/185	1.00 (0.54–1.85)^f^
				NS	Snuff (ever)	M+F	185/185	3.40 (1.00–10.90)^p^
				None	Smokeless (ever)	M+F	185/185	1.05 (0.57–1.91)^fg^
**18**	Franco 1989 [102]	Interview	Oral cavity, tongue, gum, floor of mouth	None	Smokeless (ever)	M+F	232/464	1.40 (0.59–3.33)^f^
**19**	Blomqvist 1991 [103]	Questionnaire	Lower lip	Smoking^l^	Snuff (ever)	M+F	14/10	0.67 (0.08–5.75)^f^
**20**	Maden 1992 [104]	Interview	Lip, tongue, gum, floor of mouth, oropharynx, other parts of mouth	Age	Smokeless (ever)	M	131/136	4.50 (1.50–14.30)^p^
**21**	Sterling 1992 [67]	Interview (controls), proxy interview (cases)	Oral and pharyngeal	Smoking, sex, race, age, alc, occ.	Smokeless (100+ times)	M+F	6976/NS	1.04 (0.41–2.68)^q^
				Sex, race, age	Snuff (ever)	M+F	6976/NS	2.42 (1.28–4.59)^p^
**22**	Zahm 1992 [72]	Questionnaire	Buccal cavity, pharynx	Age, time	Smokeless (ever)	M	129/248046	4.11 (2.90–5.84)^r^
**23**	Mashberg 1993 [68]	Interview	Oral cavity, oropharynx	Smoking, age, race, alc.	Snuff (ever)	M	359/2280	0.80 (0.40–1.90)^p^
					Chewing (ever)	M	359/2280	1.00 (0.70–1.40)^p^
					Smokeless (ever)	M	359/2280	0.96 (0.70–1.33)^nq^
**24**	Perry 1993^s^	NS	Oral	Smoking, age, sex, race, alc, occ.	Smokeless (100+ times)	M+F	133/678	1.43 (0.64–3.21)^q^
**25**	Kabat 1994 [59]	Interview	Tongue, floor of mouth, gums, gingiva, buccal mucosa, palate, retromolar area, tonsil and other pharynx	Smoking^l^	Snuff (ever)	M+F	195/918	4.79 (1.19–19.30)^f^
				Smoking	Chewing (ever)	M	1097/2075	1.11 (0.81–1.53)^de^
**26**	Bundgaard 1995 [105]	Questionnaire	Retromolar area, buccal mucosa, floor of mouth, hard palate, upper alveolus, lower alveolus, tongue	None	Chewing (ever)	M+F	161/400	1.44 (0.59–3.51)^d^
**27**	Lewin 1998 [64]	Interview	Oral and pharyngeal	Smoking, age, area, alc.	Snuff (ever)	M	266/641	0.98 (0.63–1.50)^r^
**28**	Muscat 1998 [106]	Interview	Major salivary gland	None	Chewing (ever)	M+F	128/114	0.89 (0.18–4.49)^d^
					Smokeless (ever)	M+F	128/114	1.19 (0.26–5.45)^dgn^
**29**	Schildt 1998 [73]	Questionnaire from next-of-kin for deceased cases/controls	Lip, tongue, gum, mouth	Smoking, alc.	Snuff (ever)	M+F	354/354	0.80 (0.50–1.30)^p^
				None	Chewing (ever)	M+F	354/354	0.60 (0.20–2.00)^p^
					Smokeless (ever)	M+F	354/354	0.87 (0.61–1.25)^fgn^
**30**	Schwartz 1998 [69]	Interview	Tongue, gum, floor of mouth, tonsils, oropharynx, other parts of mouth	Smoking, age, alc, sex	Smokeless (ever)	M	165/302	1.00 (0.40–2.30)^p^
**31**	Boffetta 2005 [82]	Questionnaire	Oral and pharyngeal	Smoking, age	Snuff (ever)	M	34/10136	1.10 (0.50–2.41)^p^
**32**	Henley 2005 [66]	Questionnaire	Oral and pharyngeal	Smoking^l^, age, race, educ., alc, exer, asp, bmi, diet, occ^t^.	Smokeless (current)	M (CPS-I)	13/77407	2.02 (0.53–7.74)^p^
					Smokeless (ever)	M (CPS-II)	46/114809	0.90 (0.12–6.71)^p^

^a ^alc = alcohol consumption, asp = aspirin, bmi = body mass index, educ = education, exer = exercise, ins = insurance status, occ = occupation

^b ^At risk population for cohort studies

^c ^OR = odds ratio (case-control studies), RR = relative risk (cohort studies)

^d ^Estimated from multiple independent 2 × 2 tables using fixed-effect meta-analysis

^e ^Numbers of exposed and unexposed cases and controls estimated from proportions exposed given numerically

^f ^Estimated from 2 × 2 table

^g ^Numbers of exposed cases and controls combined over levels of exposure

^h ^Analysis based on smokers only (only one SLT user was a non-smoker)

^i ^Numbers of cases combined over cancer types specified

^j ^Numbers of exposed and unexposed cases and controls estimated from proportions exposed given graphically

^k ^Due to the joint reporting of pharynx and larynx cases, both sites excluded from analysis

^l ^Analysis based on non-smokers only

^m ^Note that in Table 13 of source chewing only data for mouth cancer wrongly given under pipe only

^n ^Calculated assuming no-one chewed tobacco and used snuff

^o ^Larynx cases cannot be sorted out due to incomplete reporting

^p ^As reported by the authors

^q ^Estimated from non-independent relative risks by level of exposure using method of Fry and Lee

^r ^Estimated from non-independent relative risks for cancer types using method of Fry and Lee

^s ^Attributable oral cancer risk due to smokeless tobacco use based on a case-control study at Sinai Hospital in Detroit; unpublished

^t ^Occupation only adjusted for in CPS-II

Overall, Table 3 contains 53 effect estimates, of which 11 were provided by the authors and 42 were calculated from data presented. Superscript notes attached to the estimates give some detail on how the calculations were carried out. Non-independent estimates were combined over level of exposure in two studies (21, 24), over type of smokeless product in one study (23) and over cancer types in two studies (22, 27). In five studies (11, 14, 23, 28, 29) combined estimates for smokeless tobacco use were calculated approximately assuming that noone chewed tobacco and used snuff.

As described in Table 3, exposure data were collected directly from patients/controls by interview in 15 studies (2, 3, 4, 6, 7, 10, 11, 14, 18, 20, 23, 25, 27, 28, 30), by questionnaire in six (17, 19, 22, 26, 31, 32), and by both in one (5). In five studies (9, 13, 16, 21, 29), both direct and proxy interviews were conducted. In four of these, the proportion of proxy interviews was much higher in cases than in controls, and only in study 29, where controls were matched with cases on vital status, was the proportion the same. Three studies (8, 12, 15) used medical records for exposure assessment. For study 12 this may have led to the probability of reporting smokeless tobacco use being much higher in cases than in controls [10,11]. Two studies (1, 24) did not clearly describe the source of exposure assessment.

Overall 16,540 cases of oral cancer were used in the present analysis. Of these, 222 were in three cohort studies (22, 31, 32), 5,580 were in 19 case-control studies with hospital controls (1–6, 8, 10–14, 17–19, 23–25, 28), 10,704 were in nine case-control studies with population controls (9, 15, 16, 20, 21, 26, 27, 29, 30), and 34 were in the one case-control study with both types of control (study 7). The first, second and third quartiles of the number of cases considered were 94, 200, and 387. The smallest study had 14 cases (study 19) and the largest 6,976 (study 21, based on a sample of US death certificates of 1986). Excluding study 21, where numbers of controls were not available, a total of 29,009 controls were considered in the 28 case-control studies, of which 17,313 were hospital, 11,536 population and 160 mixed.

Diagnoses of oral cancer were reported as histologically confirmed in 19 studies (3, 4, 7, 8, 11, 12, 14, 16, 17, 18, 20, 23, 25, 26, 27, 28, 29, 30, 31) and as confirmed for some of the cases by four studies (9, 10, 15, 24). Histological confirmation was not reported by six studies (1, 2, 5, 6, 13, 19), and death certificates (presumably without 100% histological verification) were used in three studies (21, 22, 32).

**Figure 1:**
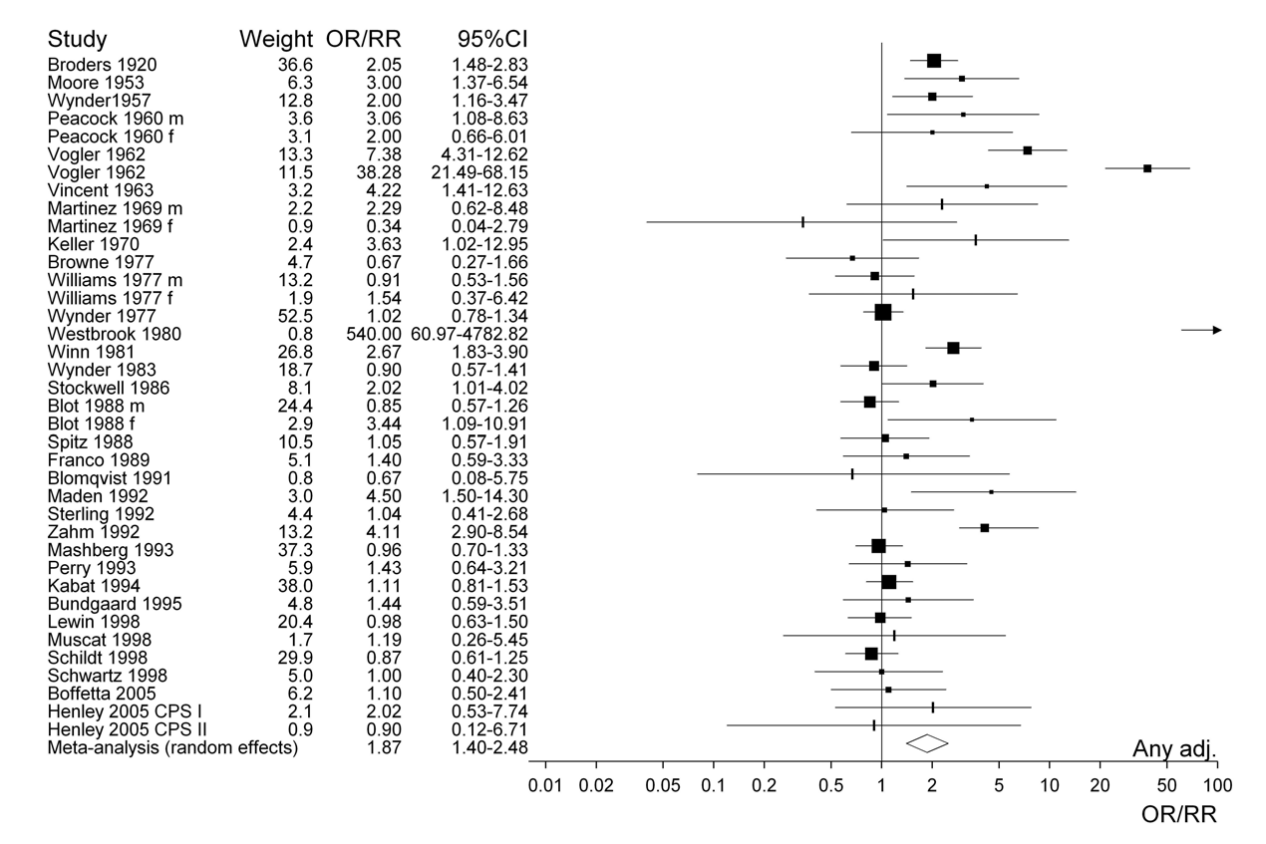
Forest plot of study-specific effect estimates and 95% CIs for all types of smokeless tobacco use.

**Figure 2:**
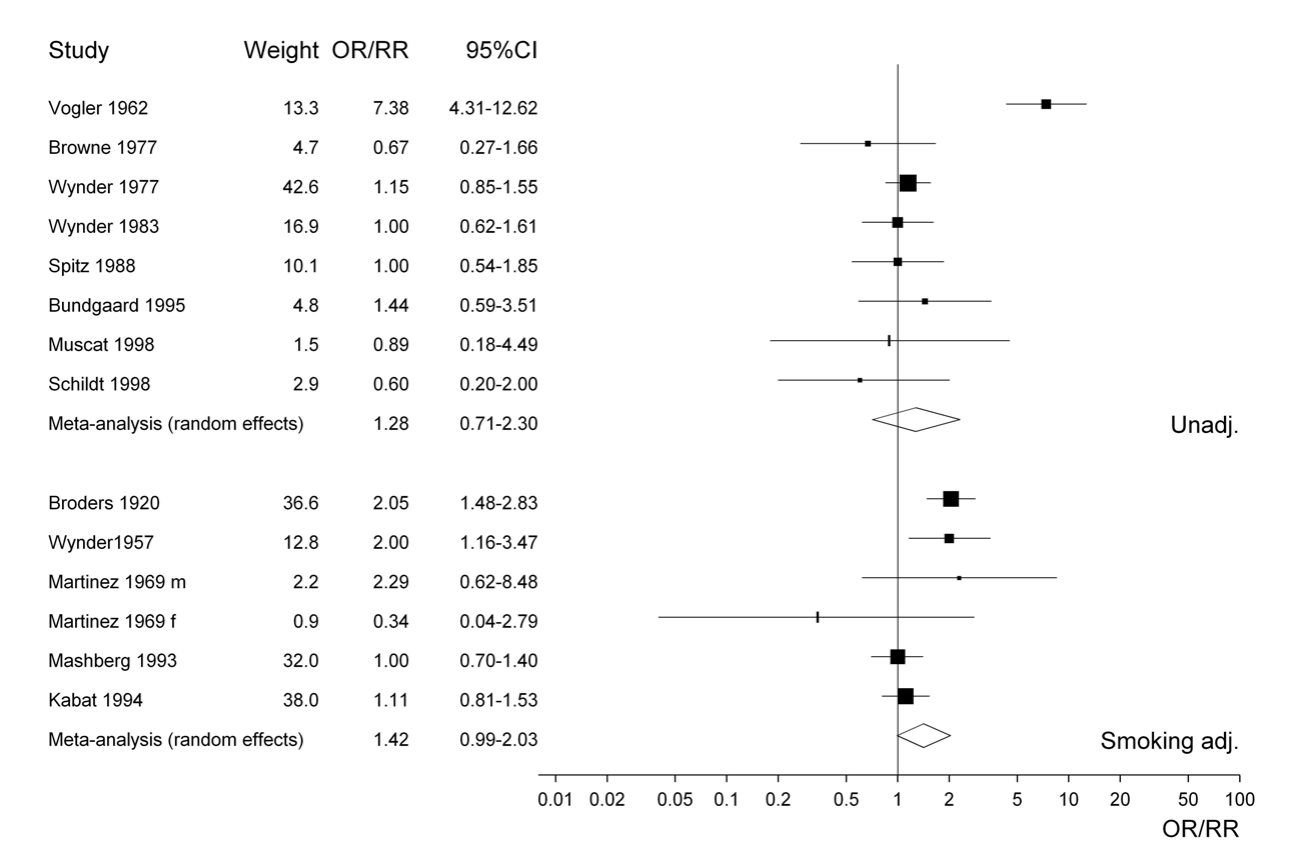
Forest plot of study-specific effect estimates and 95% CIs for chewing tobacco.

**Figure 3:**
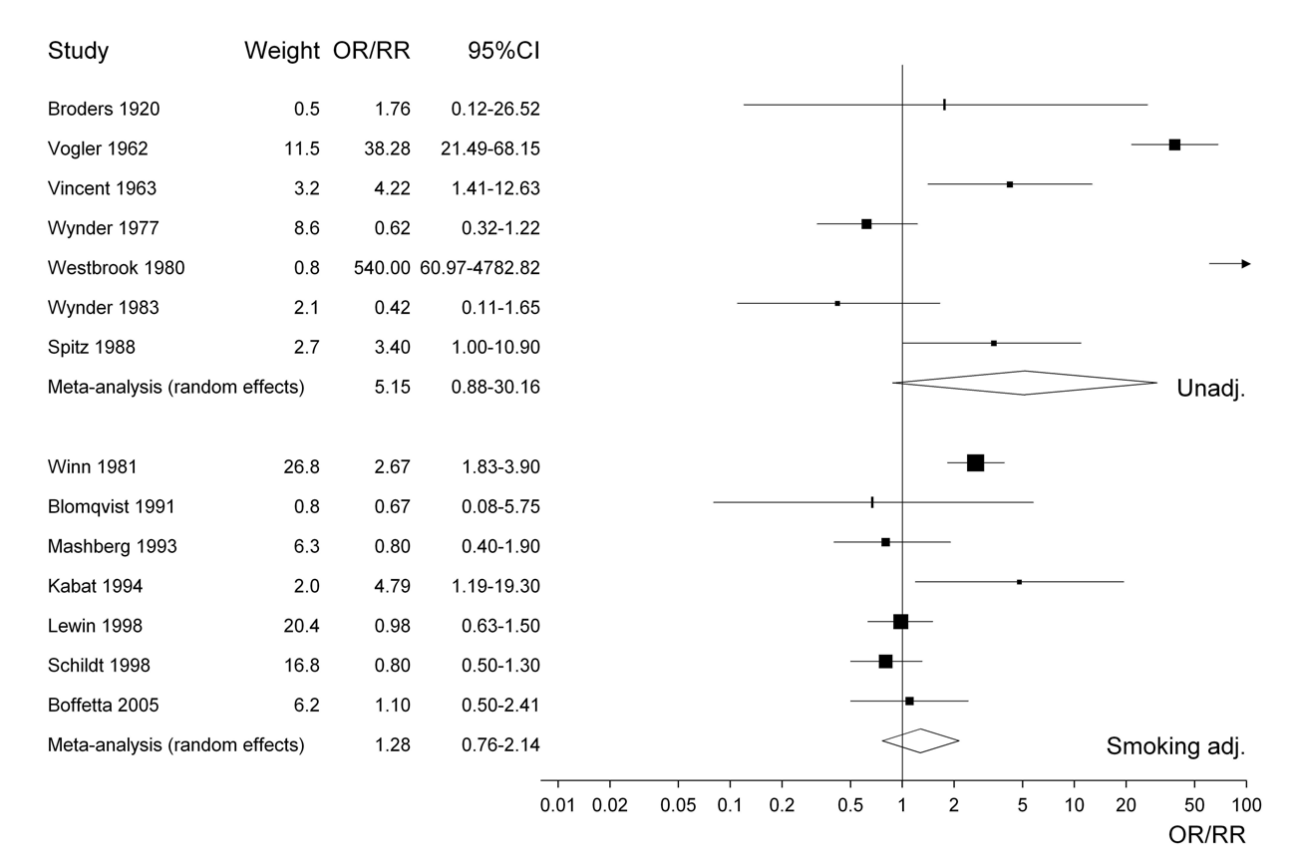
Forest plot of study-specific effect estimates and 95% CIs for snuff.

**Figure 4:**
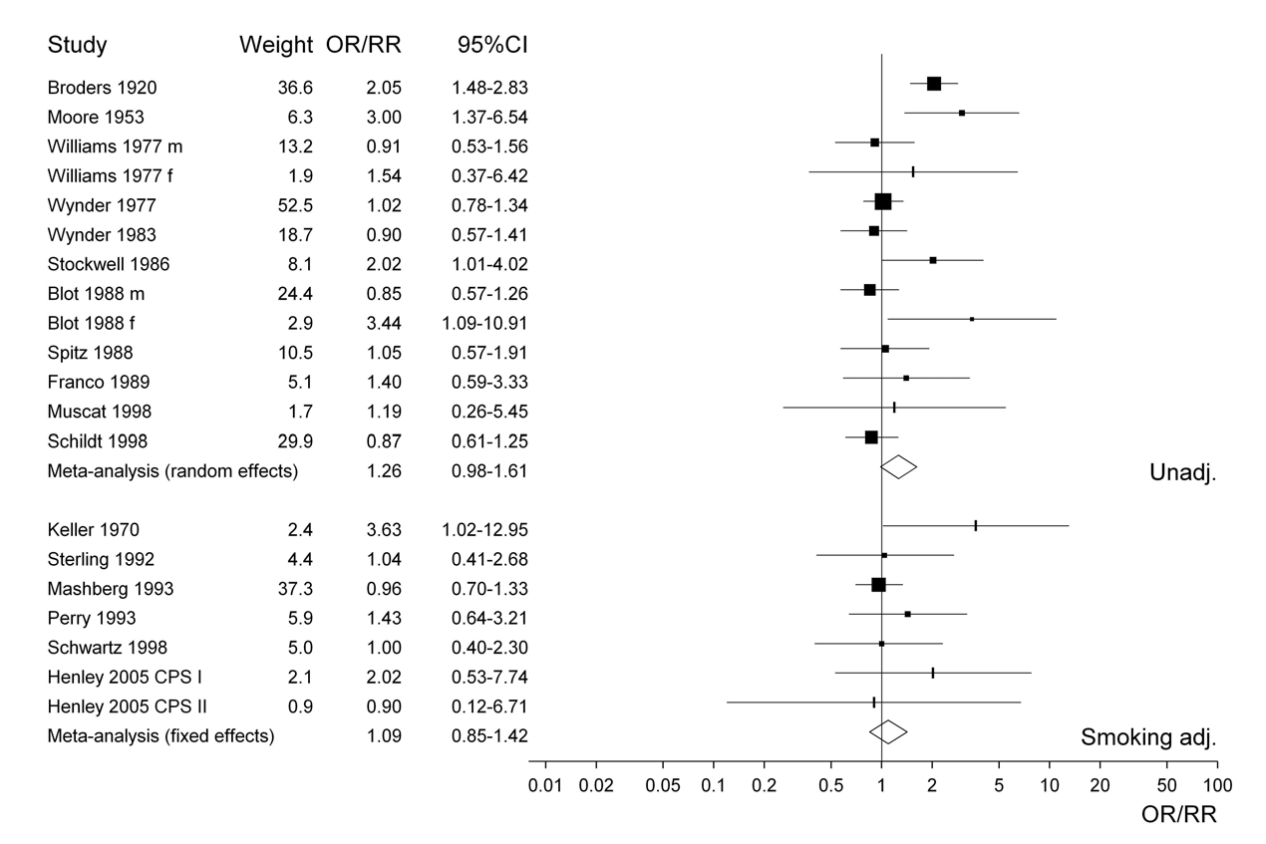
Forest plot of study-specific effect estimates and 95% CIs for overall smokeless tobacco use.

Figure 1 presents the 38 study-specific risk estimates and their 95% CI for all types of smokeless tobacco. Studies conducted before about 1965 uniformly show effect estimates above one, with studies published in the last 10 years or so showing no statistically significant increases. Figures 2 to 4 similarly present, respectively, the estimates for chewing tobacco, snuff and unspecified smokeless tobacco use. Here the estimates are separated according to whether they were unadjusted or adjusted for smoking. The precision of estimates for chewing tobacco is in general higher than the precision for snuff or unspecified smokeless tobacco use. The relatively low precision of estimates for unspecified smokeless tobacco may reflect the variety of exposures investigated. For snuff the smoking adjusted relative risks seem lower than the unadjusted relative risks, but the difference is less marked for chewing tobacco and unspecified smokeless tobacco use. The figures also include results of random-effects meta-analyses, which are discussed in the next section.

### Meta-analysis

Table 4 summarizes the results of the meta-analyses. Separate results are shown based on 14 estimates specifically for chewing tobacco, 15 specifically for snuff, 24 for smokeless tobacco, and 38 for "all" types of smokeless tobacco. Random-effects estimates appear in Table 4 only where heterogeneity was detected.

**Table 4:** Meta-analyses of the risk of oral cancer in smokeless tobacco users compared to non-users (based on the estimates given in Table 3).

					Heterogeneity		
Type of smokeless tobacco	No. of estimates combined	Adjustment factors^a^	Fixed-effect OR/RR (95%CI)	Random-effects OR/RR (95%CI)	χ^2^	I^2^	p(χ^2^)
All^b^	38	any	1.54 (1.40–1.69)	1.87 (1.40–2.48)	283.3	86.9	< 0.0001
	19	none	1.58 (1.39–1.78)	2.18 (1.35–3.53)	232.1	92.2	< 0.0001
	15	sm	1.31 (1.13–1.53)	1.35 (1.04–1.76)	28.7	51.3	0.0114
	7	sm, al	1.02 (0.82–1.28)		1.9	0.0	0.9321
	7	sm, al, age	1.02 (0.82–1.28)		1.9	0.0	0.9321
							
Chewing	14	any	1.38 (1.21–1.58)	1.36 (0.98–1.89)	61.2	78.8	< 0.0001
	8	none	1.37 (1.13–1.68)	1.28 (0.71–2.30)	46.4	84.9	< 0.0001
	6	sm	1.39 (1.16–1.66)	1.42 (0.99–2.03)	14.9	66.3	0.0110
	1	sm, al, age	1.00 (0.71–1.41)		--	--	--
							
Snuff	15	any	2.00 (1.67–2.39)	2.39 (1.17–4.89)	182.4	92.3	< 0.0001
	7	none	5.36 (3.73–7.69)	5.15 (0.88–30.2)	116.7	94.9	< 0.0001
	7	sm	1.35 (1.09–1.69)	1.28 (0.76–2.14)	24.8	75.8	0.0004
	3	sm, al	0.88 (0.65–1.18)		0.4	0.0	0.7998
	2	sm, al, age	0.93 (0.64–1.36)		0.2	0.0	0.6555
							
Smokeless^c^	24	any	1.27 (1.13–1.42)	1.46 (1.17–1.83)	65.6	65.0	< 0.0001
	13	none	1.18 (1.03–1.35)	1.26 (0.98–1.61)	31.5	61.9	0.0017
	7	sm	1.09 (0.85–1.42)		5.4	0.0	0.4967
	6	sm, al, age	1.04 (0.80–1.35)		1.8	0.0	0.8751
	4	sm, al, age, ss	1.32 (0.77–2.25)		0.8	0.0	0.8467

^a ^Smoking (sm), alcohol (al), age, social status (ss)

^b ^For each study/sex, the OR/RR for smokeless tobacco from Table 3 was included if available, otherwise that for chewing tobacco or snuff was used. For study 25 where estimates for snuff and chewing tobacco, but not smokeless tobacco, were available, that for chewing tobacco was included as it was for smokers and nonsmokers combined

^c ^This includes all the OR/RR estimates from Table 3 where the exposure was smokeless, both from studies where only estimates for unspecified exposure were available and from studies where it had been estimated from the separate data for chewing tobacco and snuff

Based on the 38 estimates for all types of smokeless tobacco shown in Figure 1, the fixed-effect estimate, regardless of adjustment for potential confounding variables, is 1.54. This is similar to the fixed-effect estimate of 1.58 based on studies without any adjustment. In both cases, heterogeneity is observed, substantial enough to cast some doubts on the corresponding random-effects estimates.

When only those estimates adjusted for smoking are considered, the heterogeneity is markedly reduced, and the fixed-effect estimate also reduces, to 1.31. Since significant heterogeneity remains, the random-effects estimate of 1.35 is the preferred overall estimate. When the analysis is further restricted to estimates adjusted for both smoking and alcohol consumption, no heterogeneity is seen. Moreover, the point estimate is close to unity (1.02; 95% CI 0.82–1.28). The lack of detectable risk when these factors are accounted for seems consistent with an important confounding effect of smoking and alcohol, both well known major risk factors for oral cancer. For example, Döbróssy [61] reports that smoking and alcohol consumption each "may account for a two- to three-fold increase in risk" and "when tobacco smoking and alcohol consumption are combined, they may increase the risk by more than 15-fold."

The same pattern of estimates decreasing after adjustment for smoking and alcohol can be seen from the separate results for chewing tobacco, snuff and unspecific smokeless tobacco, although for snuff and particularly chewing tobacco numbers of estimates adjusted for both factors are very low. None of the analyses show any significant increase in risk following adjustment for both factors.

The elevated random-effects estimate 5.15 for snuff based on unadjusted data (see also Figure 3) is mainly due to two studies with evident weaknesses [62,63]. The study by Vogler et al. [62] did not match controls to cases and the effect estimate of 38.28 (21.49–68.15) was computed based on rather incompletely reported information. With regard to the study by Westbrook et al. [63], both IARC[10] and the US Surgeon General [11] pointed out that the risk estimate of 540.0 (60.97–4782.82) is very unreliable, exposure assessment being based on hospital records, where documentation of smokeless tobacco use is much more likely in cases than in controls. Omitting these two studies, the unadjusted random-effects estimate for snuff falls to 1.40 (0.51–3.86), with the I^2 ^value reduced from 94.9 to 71.6.

### Sensitivity analyses

Of the 38 estimates included in the all smokeless tobacco analyses, six (from studies 3, 7, 19 and 32) were for smokers or for nonsmokers. Eliminating these estimates so that all the data were for smokers and nonsmokers combined reduced the fixed-effect estimate only slightly, from 1.54 (1.40–1.69) to 1.53 (1.39–1.68) but increased the random-effects estimate from 1.87 (1.40–2.48) to 1.94 (1.43–2.65).

Of the 38 estimates, five (from studies 11, 14, 23, 28 and 29) were derived approximately assuming that there were no joint users of chewing tobacco and snuff. Replacing these by estimates for chewing tobacco in US studies and for snuff in Swedish studies increased the fixed-effect estimate to 1.62 (1.47–1.78) but left the random-effects estimate the same at 1.87 (1.40–2.50).

### Heterogeneity

Of the 38 estimates used in the all smokeless tobacco analyses, only five were provided directly in the publications, the remaining 33 being derived from the data available. Although fixed-effect estimates were lower for the published data (1.21; 0.78–1.89) than for the derived data (1.55; 1.41–1.71), the difference was not statistically significant (χ^2 ^= 1.15, p = 0.28). Although the number of published estimates is too few for reliable interpretation, this suggests that no bias to the null has been introduced by considering derived estimates.

A more detailed heterogeneity analysis was also undertaken (Table 5). Since the number of estimates for specific types of smokeless tobacco (14 for chewing tobacco and 15 for snuff) was rather small, and subdivision for possible sources of heterogeneity would have resulted in various cells containing at most a single study, this analysis was conducted using the estimates included in the all smokeless tobacco analyses. Since there were few estimates that were adjusted for both smoking and alcohol consumption, and few adjusted estimates which did not include smoking as one of the factors adjusted for, the heterogeneity analyses were conducted based firstly on the 19 estimates that were unadjusted for any factor and second on the 15 estimates that were adjusted for smoking, regardless of any other adjustment. This left a reasonable number of individual estimates in each of the two sets to investigate the possible role of type of smokeless tobacco, sex, study design, study location and study period on the estimated effect.

**Table 5:** Heterogeneity of the oral cancer smokeless tobacco relative risk, unadjusted and adjusted for cigarette smoking (based on the estimates given in Table 3).

	Unadjusted for any factor					Adjusted for smoking				
			Heterogeneity^b^					Heterogeneity^b^		
Factor	N	OR/RR (95%CI)^a^	χ^2^	I^2^	p(χ^2^)^c^	N	OR/RR (95%CI)^a^	χ^2^	I^2^	p(χ^2^)^c^

**All estimates**^d^	19	1.58 (1.39–1.78)	232.1	92.2	< 0.0001	15	1.31 (1.13–1.53)	28.7	51.3	0.0114
**Type**										
*Chewing tobacco*	3	3.20 (2.12–4.82)	23.8	91.6	< 0.0001	4	1.29 (0.99–1.69)	5.6	46.2	0.1342
*Snuff*	3	27.9 (17.0–45.9)	19.6	89.8	0.0001	4	1.62 (1.24–2.11)	13.4	77.7	0.0038
*Smokeless*^e^	13	1.18 (1.03–1.35)	31.5	61.9	0.0017	7	1.09 (0.85–1.42)	5.4	0.0	0.4967
***Between levels***			**157.3**	**67.7**	**< 0.0001**			**4.3**	**15.1**	**0.1142**
**Sex**										
*Female*	4	20.3 (12.6–32.5)	35.0	91.4	< 0.0001	2	2.51 (1.73–3.64)	3.5	71.5	0.0610
*Male*	7	1.27 (1.07–1.51)	60.6	90.1	< 0.0001	10	1.15 (0.97–1.37)	10.8	16.4	0.2925
*Both*	8	1.35 (1.11–1.64)	16.5	57.5	0.0213	3	1.19 (0.66–2.15)	0.6	0.0	0.7573
***Between levels***			**120.1**	**51.7**	**< 0.0001**			**13.9**	**48.4**	**0.0010**
**Study design**										
*Case-control, hospital controls*	13	1.90 (1.64–2.21)	200.4	94.0	< 0.0001	7	1.41 (1.18–1.68)	22.0	73.7	0.0009
*Case-control, population controls*	6	1.01 (0.81–1.27)	11.0	54.6	0.0514	3	0.99 (0.69–1.42)	0.0	0.0	0.9935
*Case-control, mixed controls*	0	--	--	--	--	2^f^	1.35 (0.44–4.13)	--	--	0.1337
*Cohort*	0	--	--	--	--	3	1.24 (0.65–2.36)	--	--	0.7062
***Between levels***			**20.7**	**8.9**	**< 0.0001**			**3.0**	**10.4**	**0.3928**
**Study location**										
*USA*	15	1.76 (1.54–2.02)	215.6	93.5	< 0.0001	10	1.39 (1.17–1.64)	23.7	62.1	0.0047
*Scandinavia*	2	0.93 (0.67–1.30)	1.1	5.3	0.3041	3	0.99 (0.68–1.45)	0.2	0.0	0.9054
*Other*	2^g^	0.99 (0.53–1.84)	1.3	24.6	0.2495	2^h^	1.35 (0.44–4.13)	2.2	55.5	0.1337
***Between levels***			**14.2**	**6.1**	**0.0008**			**2.5**	**8.8**	**0.2810**
**Study period**										
*-1969*	5	4.49 (3.55–5.66)	79.8	95.0	< 0.0001	4	2.02 (1.28–3.20)	3.6	15.8	0.3129
*1970–1979*	5	1.05 (0.84–1.33)	33.0	87.9	< 0.0001	1	2.67 (1.83–3.90)	0.0	0.0	--
*1980–1989*	7	1.01 (0.83–1.23)	10.4	42.4	0.1080	2	0.97 (0.71–1.31)	0.0	0.0	0.8743
*1990+*	2	1.37 (0.64–2.96)	0.0	0.0	0.8321	8	1.10 (0.88–1.37)	1.8	0.0	0.9719
***Between levels***			**108.9**	**46.9**	**< 0.0001**			**23.4**	**81.4**	**< 0.0001**

^a ^Fixed-effect estimates

^b ^For the "Between levels" rows, the I^2 ^column contains the percentage of variation explained by the factor levels rather than the heterogeneity I^2 ^statistic

^c ^The probability for the heterogeneity χ^2 ^between studies within levels is shown, except that for the "Between levels" rows, the probability is for the between levels χ^2^

^d ^For each study/sex, the OR/RR for smokeless tobacco from Table 3 was included if available, otherwise that for chewing tobacco or snuff was used. For study 25 where estimates for snuff and chewing tobacco, but not smokeless tobacco, were available, that for chewing tobacco was included as it was for smokers and nonsmokers combined

^e ^This includes all the OR/RR estimates from Table 3 where the exposure was smokeless, both from studies where only estimates for unspecified exposure were available and from studies where it had been estimated from the separate data for chewing tobacco and snuff

^f ^In study 7 (two estimates), both hospital and population controls were used

^g ^Studies 9 (UK) and 18 (Brazil)

^h ^Study 7 (conducted in Puerto Rico, two estimates)

^I ^Since only one study with an effect estimate adjusted for smoking was conducted between 1970 and 1979 (study 13), an additional assessment of heterogeneity was carried out based on two periods (-1979 vs. 1980+). The corresponding overall effect estimates, based on 5 and 10 studies respectively, were 2.39 (1.78–3.19) and 1.05 (0.88–1.26), with p < 0.0001 for between levels heterogeneity

Since the potential sources of heterogeneity must be considered as competing, and because of possible residual confounding, for example due to alcohol consumption, the analyses probably overestimate the true contributions to heterogeneity of the individual factors assessed. True proportions of explained heterogeneity could only be estimated from multifactorial analyses of primary data.

By far the most profound source of heterogeneity was study period. In smoking-adjusted estimates, 81.4 percent of the total heterogeneity was accounted for by between-period heterogeneity. Studies conducted before 1980 showed an excess risk, while no increase was seen from studies conducted in 1980 or later. In smoking-unadjusted estimates, period also accounted for a large proportion (46.9%) of the total heterogeneity.

Although 67.7% of the total heterogeneity could be accounted for by type of tobacco in the unadjusted analyses, the corresponding proportion for the smoking-adjusted risk estimates was only 15.1%. Although the smoking-adjusted effect estimates for chewing tobacco (1.29) and snuff (1.62) were elevated, this may reflect uncontrolled confounding by alcohol as no increase was seen in the analyses in Table 4 based on estimates adjusted for smoking and alcohol.

Studies conducted in the US indicated an increased risk of oral cancer in both unadjusted data (by 76%) and adjusted data (by 39%). Though no significant effect was seen in Scandinavia or in other countries, numbers of estimates were relatively few (2 in Scandinavia, 1 in UK and 1 in Brazil for the unadjusted data; 3 in Scandinavia, 2 in Puerto Rico for the adjusted data), and heterogeneity by location was only demonstrated for the unadjusted data.

Sex-specific overall risk estimates were higher for women than men. The fact that the sex difference was larger using unadjusted estimates suggests that at least part of it may be due to confounding.

Hospital-based case-control studies provided higher risk estimates than those from other study designs, and their estimates were much more heterogeneous. The reason for this is unclear.

**Figure 5:**
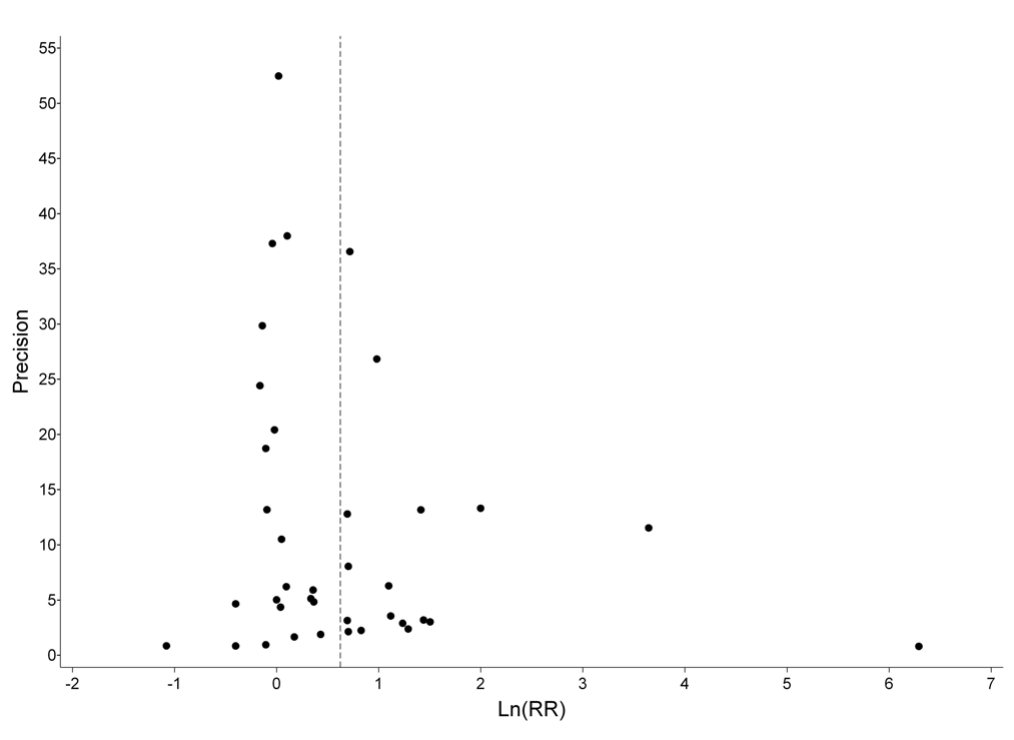
Funnel plot of 38 study-specific effect estimates against precision (1/variance of log effect estimate).

Figure 5 plots all study-specific effect estimates on a logarithmic scale against their precision. There is only a slight inverse association between precision and variability of individual estimates around the overall random-effects estimate of 1.87 (dotted vertical line). The observed asymmetry of the funnel plot suggests that publication bias cannot be excluded.

### Never smokers

While the previous analyses are based on results, where available, for the whole population studied, including smokers and never smokers, six studies provided oral cancer relative risks specifically among never smokers. The estimates, two for snuff use and four for general smokeless tobacco use, are summarized in Table 6, as are meta-analysis results. Numbers of exposed cases are extremely low, only 44 in total, and six or less for all but one of the estimates. Although the data are somewhat suggestive of an association, neither the fixed-effect estimate of 1.30 (95%CI 0.82–1.93) nor the random-effects estimate of 1.94 (0.88–4.28) is statistically significant and the data are heterogeneous (I^2 ^= 63.1). Also shown in Table 6 is an additional age- and region-adjusted estimate of 4.70 (1.60–13.80) for ever snuff use in males. This estimate is for oral, larynx and oesophagus cancer combined, so does not satisfy our original inclusion criteria. Were it included, the random-effects estimate would become significant, at 2.20 (1.04–4.67).

**Table 6:** Effect estimates for never smokers^a^

**Study**	**First author, year of publication**	**Adjustment factors**	**Exposure**	**Sex**	**Exposed cases**	**OR/RR (95%CI)**
Studies providing result specific for oral cancer						
8	Keller 1970 [75]	None	Smokeless	M	4	3.04 (0.62–14.99)
16	Blot 1988 [100]	Age, race, location, respondent	Smokeless	F	6	6.20 (1.90–19.80)
19	Blomqvist 1991 [103]	None	Snuff	M+F	2	0.67 (0.08–5.75)
25	Kabat 1994 [59]	None	Smokeless	M	4	1.59 (0.51–4.96)
				F	4	38.7 (2.1–723.6)^b^
29	Schildt 1998 [73]	Age, sex, residence	Snuff	M+F	19	0.70 (0.40–1.20)
32	Henley 2005 [66]	Age, race, education, alcohol, exercise, aspirin, body mass index, diet, occupation^c^	Smokeless	M (CPS-I)	4	2.02 (0.53–7.74)
				M (CPS-II)	1	0.90 (0.12–6.71)

		Fixed-effect meta-analysis estimate for six studies				1.30 (0.87–1.93)
		Random-effects meta-analysis estimate for six studies				1.94 (0.88–4.28)
		Heterogeneity	χ^2 ^(df)			18.99 (7)
			p			0.0082
			I^2^			63.13

Additional study providing result for oral, larynx and oesophagus cancer combined						

27	Lewin 1998 [64]	Age, region	Snuff	M	9	4.70 (1.60–13.80)

		Fixed-effect meta-analysis estimate for all seven studies				1.51 (1.04–2.19)
		Random-effect meta-analysis estimate for all seven studies				2.20 (1.04–4.67)
		Heterogeneity	χ^2 ^(df)			23.82 (8)
			p			0.0025
			I^2^			66.42

^a^See the discussion section for the reasons why data from study 15 [65] were not included in Table 6

^b ^There were no exposed controls and the OR was estimated by adding 0.5 to each cell in the 2 × 2 table

^c^Occupation only adjusted for in CPS-II

## Discussion

This report is based on data relating oral cancer risk to the consumption of chewing tobacco and snuff as used in Western countries. Our main analyses, based on results for smokers and nonsmokers combined where possible, considers 53 individual effect estimates based on 32 studies published from 1920 to 2005. This represents 11 more studies than considered in the previous most recent quantitative review of the evidence[16]. 16,540 cases of oral cancer were included in studies of different design, size, and quality. Many of the 32 reports have limitations and present less information than is ideal for a meta-analysis. Shortcomings include small numbers of cases (particularly exposed cases), lack of histological confirmation, lack of division by cancer site, as well as an unclear description of inclusion and exclusion criteria, details of case and control selection, and methods of exposure assessment. Furthermore, exposure details such as type of smokeless tobacco, duration and frequency of use were often not considered. At the analysis level the main weaknesses were failure to adjust results for important potential confounders and to present results separately for major subgroups, particularly by sex, smoking and alcohol. These weaknesses inevitably limit the inferences that can be drawn. Nevertheless some conclusions can be drawn from the present results.

Apart from the diversity of designs, samples, procedures, methods, and types of smokeless tobacco investigated, the individual effect estimates were themselves highly variable, particularly for snuff, where the I^2 ^statistic for the 15 estimates was 94.9 (see also Figure 1). Given the well-known strong relationships of smoking and alcohol consumption to oral cancer risk [52], and given that 33 of the 53 estimates were unadjusted for either smoking or alcohol, it was perhaps unsurprising that this heterogeneity was highly reduced, and indeed essentially eliminated (I^2 ^= 0 for each of the types of smokeless tobacco) by restricting attention to estimates adjusted for these two variables. Not only did such adjustment remove the substantial heterogeneity, but it removed the association, with an overall estimate for all smokeless tobacco use of 1.87 (1.40–2.48) reducing to 1.02 (0.82–1.28). The latter estimate is consistent with risk of oral cancer being independent of smokeless tobacco use, though a small increase cannot of course be totally excluded.

The lower effect estimates seen in studies that adjust for alcohol and smoking need not wholly be due to the adjustment. For example, unadjusted estimates may derive from studies which are methodologically weaker, perhaps because smokeless tobacco was not a central issue. As such it is important to look at other information which may cast light on the role of confounding by alcohol or smoking. One way of attempting to eliminate potential confounding effects of smoking is to restrict attention to study of the effects of smokeless tobacco use in never smokers. Unfortunately only six studies provided effect estimates for oral cancer or data from which these could be calculated. Although the data are suggestive of an association, the meta-analysis estimates (see Table 6) are inconclusive and the data are heterogeneous. They are also no doubt open to publication bias, since many researchers would not present null results based on very limited data. These data involve very few exposed cases. Any data available for subjects who have never smoked and never used alcohol would be even sparser. As can be seen from Table 6, one additional study [64] provided results that were not for oral cancer specifically, but included other forms of head and neck cancer. Inclusion of these results do not affect the above conclusions. Another study [65] also seemed at first glance to provide possibly relevant results, with significant increases noted for some oral sites and not others. However this study only asked about the primary type of tobacco used, comparing smokeless tobacco users, who may well also have smoked, with subjects who never used any tobacco product at all. These results are clearly not restricted to never smokers and are open to confounding.

To further assess possible effects of adjustment, it is helpful to consider in more detail those studies which provide both adjusted and unadjusted estimates. Lewin et al. [64] saw no effect of snuff before or after adjustment for alcohol and smoking, but did observe a clear dose-response relationship both with alcohol and smoking. Henley et al. [66] found that the non-significant association of oral cancer with smokeless tobacco use seen in never smokers was further reduced after adjustment for alcohol consumption. Perry et al. (Attributable oral cancer risk due to smokeless tobacco use based on a case-control study at Sinai Hospital in Detroit; unpublished) found that additional adjustment for alcohol and smoking reduced risk estimates adjusted only for sex, race, and age from 2.51 to 1.86 and from 1.30 to 0.93 in groups with, respectively lower and higher smokeless tobacco use. Sterling et al. [67] found that a significant sex, race, and age-adjusted association (2.42) in ever as compared to never smokeless tobacco users was virtually eliminated after additional adjustment for alcohol consumption, smoking and occupation. These authors emphasized the strong dose-response for alcohol consumption (as seen also in other studies considered [68,69]) as compared to the lack of association for smokeless tobacco use. Although the evidence is limited, the findings are consistent with the notion that confounding by smoking and particularly by alcohol consumption, clearly shown to be a major risk factor in oral cancer [70], is an important consideration.

Unfortunately, the number of estimates adjusted for both smoking and alcohol consumption was rather limited. Overall estimates of the risks associated with chewing tobacco and snuff which can reasonably be compared with each other could therefore be based only on those study-specific estimates (six and seven, respectively) which were adjusted at least for smoking. However, the corresponding estimates of 1.42 (0.99–2.03) and 1.28 (0.76–2.14) have quite low precision and provide little useful information on whether the associations truly differ. Anyway, they are likely to be biased upward by uncontrolled confounding by alcohol.

For confounding to occur it is necessary for smokeless tobacco users and non-users to differ in their smoking and alcohol consumption. However relevant data seem quite limited and somewhat variable. The evidence seems more consistent for smoking where data indicating a positive relationship with smokeless tobacco use are seen in published studies in the USA [59,71,72] and in Sweden [73] and also in unpublished data provided by Statistics, Sweden (I Sjöberg, personal communication). For alcohol consumption, one study in Sweden [73] and one in the US [43] report a strong association, consistent with the possibility of substantial confounding, but such an association is not clearly evident in other studies. Thus data for men in CPS I and CPS II [66] and from the first National Health and Nutrition Examination Survey [NHANES I][41,71] show only a weak association, and, in women, reported alcohol consumption in NHANES I is clearly lower in smokeless tobacco users than in non-smokeless tobacco users, both in smokers and nonsmokers. In a study in North Carolina Winn et al. [13] reported that "most women who dipped snuff did not smoke cigarettes or consume alcoholic beverages," though Nilsson [14] suggested that alcohol consumption may not have been considered proper behaviour for women when the study was conducted, and that this may have led to it being substantially underreported. Whether smokeless tobacco users are more likely to underreport alcohol consumption is unclear.

The importance of confounding by alcohol consumption cannot, however, be fully resolved from the data available. Not only are the available data on the association of alcohol and smokeless tobacco very limited, they are unadjusted for age and race and usually for smoking or sex. Anyway the association may vary by type of smokeless tobacco, type of alcohol, country and period. It certainly seems to us that, at least in some studies, observed differences in risk of oral cancer associated with use of smokeless tobacco and type of tobacco used may be due to confounding by alcohol. In this context it should be noted that other potential sources of confounding are not even mentioned by most authors, like exposure to sunlight (being relevant for lip cancer) which might (as might the use of smokeless tobacco) show a social gradient due to an association with the type of occupation.

Although conclusions based on adjusted estimates seem more convincing than those based on unadjusted ones, they are still not very reliable. One reason is the small number of available estimates. A second reason is the limited nature of the adjustment – thus many of the studies that adjusted for smoking did not take into account amount smoked (e.g. [13,74,75]) and one of those that did [68] combined never and light smokers into a single category. Simple adjustment for smoking in broad groups, e.g. never/former/current, may bias risk estimates for smokeless tobacco downwards if in fact smokers who also use smokeless tobacco smoke fewer cigarettes than smokers who do not. The evidence here seems somewhat conflicting, with some studies [73,76] reporting similar cigarette consumption in the two groups, two studies [77,78] reporting somewhat lower consumption in the smokers who also use smokeless tobacco, and one study [41] reporting somewhat higher consumption (here measured by pack-years). Though it would clearly have been better had all studies adjusted for daily cigarette consumption, any bias from this source is likely to be modest. Adjusting for whether or not the subject is a smoker seems to be of more importance.

A third reason for unreliability of the adjusted estimates is the variability of the findings. As the heterogeneity analysis revealed, study period was a major source, with smaller risks found in more recent studies. In fact, those studies which reported very large relative risk estimates for specific sites [13,62,63] are rather old. Despite methodological limitations, the strength of these site-specific effects indicates that use of oral snuff in the USA at that time was associated with an increased oral cancer risk, especially at locations where the snuff was held. The secular decline in relative risk is encouraging and suggests that even if current use of Western smokeless tobacco poses some risk, it is substantially less than it was decades ago. This decline, and also the decline in US oral cancer rates, may relate to the fact that levels of tobacco-specific nitrosamines [TSNAs] in American smokeless tobacco products, historically much higher than in their Swedish counterparts, have declined by more than 70 percent in the last 25 years [52,79]. However we note that there is no definitive evidence linking TSNAs in smokeless tobacco products with oral cancer risk, and that the available data are insufficient to take into account reliably the time lag between smokeless tobacco use and possible development of oral cancer.

Our analyses of smoking adjusted risks seemed to suggest that, compared to non-users, female users of smokeless tobacco might be at higher risk than male users, with fixed-effect relative risks of 2.51 vs 1.15. The result for females is, however, based on only two studies, of which only the Winn et al. study [13] found an excess risk. Random-effects estimates are similar for women (1.25) and men (1.19), and though one cannot rule out the possibility of an increased effect in women, one cannot infer this from the limited data.

Our analyses showed some indications of publication bias, a finding supported by others [80]. Specific indices of publication bias can also be found in the literature we studied. For example in two studies [32,33] negative results were reported, but no details given.

Our review is consistent with a general trend in appraising the potential risk of oral cancer from use of smokeless tobacco. In the mid 1980s smokeless tobacco was assessed as a risk factor for oral cancer by IARC and the US Surgeon General [10,11]. Although, according to an advance report on a forthcoming monograph [81] IARC seems to maintain this view, more recent reviews have reached different conclusions. Whereas tobacco chewing seems to be a major risk factor for oral and pharyngeal cancer in Asia [7] it is now considered unlikely to incur a substantial risk among users of smokeless tobacco products in the United States or Europe (e.g.[82]). The difference in risk between Western smokeless tobacco products and those used in developing countries may be related to tobacco species, fermentation and ageing [83]. Also, the addition of ingredients other than tobacco like betel quid, ash and lime might play a role[12]. For Western tobacco, various reviewers [84-86] have emphasized that any risk of life-threatening diseases in general that is associated with smokeless tobacco use is very much less than that associated with smoking. For example, Bates et al. [84] conclude that "on average Scandinavian or some American smokeless tobaccos are at least 90% less hazardous than cigarette smoking."

These reviews have led to discussion as to whether smokeless tobacco might be a less dangerous alternative to smoking for those who do not quit [87-89]. Unlike in the US, tobacco for oral use has been banned in all EU countries, except for Sweden, and a ban also exists in Switzerland. Fagerström and Schildt [90] refer to the low (and declining) prevalence of smoking and the high (and increasing) use of smokeless tobacco in Sweden, and suggest this may be responsible for Sweden having the lowest incidence of tobacco-related disease among developed countries (see [89]). Fagerström and Schildt [90] report that 47% of current snus users were former smokers and 28% of ex-smokers used snus at their last attempt to stop smoking. An effect of snus use on smoking cessation was also suggested by Gilljam & Galanti [91], who found that the proportion of Swedish men that had ever used snus was larger in former than current smokers (55 vs. 45%). Discussing whether snus might be a gateway to smoking, Fagerström and Schild [90] report that only 6% of daily smokers had started tobacco consumption with snus. They concluded that "on balance, there is reason to believe that having snus available to the Swedish population has been of benefit to public health." Whether or not smokeless tobacco use can play a role in helping smokers quit and reducing population risk overall has been fiercely debated in recent years (e.g. [76,78]).

## Conclusion

Detailed assessment of the overall risks and benefits of smokeless tobacco use to public health requires consideration of the whole spectrum of its possible health effects and is beyond the scope of this review. While it is clear that there are unique risks from smokeless tobacco use (notably on non-malignant lesions of the oral mucosa [92]), there are numerous reports that support the risks of smoking-related diseases from smokeless tobacco as being generally much less than those from smoking, as was noted above. Although we deliberately refrain from concluding here that smokeless tobacco products represent a lower overall disease risk alternative to cigarettes, we do conclude from our findings that the available data suggest at most a minor increased risk of oral cancer associated with the use of a wide range of currently used Western chewing tobacco and snuff. Observed associations may well be due to confounding by smoking or alcohol consumption. Any effect that increasing use of smokeless tobacco use might have at the population level on risk of oral cancer may depend more on the effect such an increase might have on smoking or alcohol consumption than on any direct effect of smokeless tobacco itself.

## Competing interests

ES and RW work for Philip Morris International (PMI), R&D. Both receive their salary from PMI and both own shares in Altria, the holding company of PMI. PNL, founder of P.N. Lee Statistics and Computing Ltd., is an independent consultant in statistics and an advisor in the fields of epidemiology and toxicology to a number of tobacco, pharmaceutical and chemical companies.

## Authors' contributions

PNL conducted an unpublished review and meta-analysis on this subject in 2002. Although RW conducted and wrote up the present analyses, the earlier work provided an extremely helpful basis for comparison and validation at various stages. PNL also assisted with the preparation and layout of the final manuscript for publication. ES initiated the work and made substantial contributions to planning, analysis and writing. He continuously contributed to the work, revised the text critically, and also provided the final language corrections. All authors read and approved the final manuscript.

## Pre-publication history

The pre-publication history for this paper can be accessed here:


